# Supplementary material for: How statistical learning interacts with the socioeconomic environment to shape children’s language development
Source: PLoS One. 2021 Jan 22;16(1):e0244954. doi: 10.1371/journal.pone.0244954 (PMC7822340; doi:10.1371/journal.pone.0244954)
Supplement: Attachment — Submitted filename: PONE-D-19-17535_R&R_072419.docx [file pone.0244954.s001.docx]

What a fantastic and timely paper! I am very excited about the findings presented in this manuscript. The authors make an excellent case for why this research is necessary, and the argument for investigating the interaction SES and serial pattern learning have upon language development is strong. I also greatly appreciate how straight forward and clear the writing is. That being said, I have some questions related to how the EEG data was analyzed that will potentially require major revision on the part of the authors.

**Major:**

General:

There are reports of a MMN difference for low versus high probability items in statistical learning paradigms/auditory oddball paradigms. Additionally, there are reports of N400/ERAN differences in similar paradigms. Given that other components could be present that could inform us of additional cognitive processes at play during learning, I am curious why the authors have focused their analysis only on the P300 component?

Introduction:

Lines 65-66: “Alternatively, it is possible that learning ability cannot compensate for such environmental limitations.” Do the authors feel it is possible that environmental factors could influence learning ability early on? For example, coming from a low SES home, where you receive poorer quality/quantity input, or poorer nutrition/higher stress/etc., could influence pattern learning?

Can the authors give a more detailed account of what serial pattern learning ability is, including background literature that links this specific type of learning to language development (with specific examples)?

Additionally, can the authors include more concrete examples of how the P300 has been shown to reflect learning in similar paradigms? Specifically, discussing the previous studies (referenced in line 149) that lead the authors to predict faster reaction times and larger ERPs for HP than LP predictors.

Methods:

Measures:

Can the authors please include a breakdown of how many children were in each age group? Additionally, what was the mean age for each group in Table 1 (i.e. did age significantly differ across SES strata)?

EEG:

What was the sampling rate at collection? Was the data re-sampled?

Was the data low-pass/high-pass filtered?

What was the reference electrode during collection? Was the data re-referenced offline?

How were segments of bad data removed? The authors state these were removed manually, but this occurs at the end of their EEG section, so did they remove them after already epoching the data and running ICA?

The segment (epoch) length does not make sense to me. As I understand it, the stimuli was presented for 500 msec followed by a 500 msec black screen. This allows for a 1000 msec window per stimulus. Why would the authors epoch length then be 1500 msec with a 200 msec baseline? This includes the next predictor in the epoch—correct? Even if the authors are only going to investigate the first 500 msec of the epoch, additional noise is being introduced. If my interpretation is correct, I unfortunately would strongly recommend they epoch the data in a window that is more appropriate to their stimulus and time window of interest (-200 to 800 msec or something similar).

When the authors state that data were “replaced with data induced from surrounding sensors using the computational MATLAB software”, does this mean data was interpolated? Was this a spherical interpolation? Some other method?

How were ERPs created? Specifically, was the data low-pass filtered a second time? Was the data baseline corrected? Were single trials averaged together to obtain a stable waveform? Was this done for each condition and each electrode for every subject?

Results:

I was surprised to see the authors analyzed the time window of 400-700 msec, as the P300 is traditionally 250-500 msec. Can this be considered the same effect?

I greatly appreciate the authors inclusion of all data in Figure 4; however, this emphasizes to me that there are additional effects at play. A data driven approach, involving a cluster analysis (or some other variation where all time windows and electrodes are considered), would better allow for the investigation of all these effects. That being said, if the authors can make a stronger argument for a) only looking at the P300, b) only focusing on the 6 electrodes of interest and c) the time window of interest, I can also appreciate analyses that are rooted in solid apriori hypotheses. At this time though, those hypotheses (or why these choices were made) are not as clear as they need to be to not warrant a stronger, more data-driven approach.

Thank you for the Bonferroni corrections and for the inclusion of order (which turned out to be significant).

Correlation analyses: Given that the age range is very broad (7-12) and amplitude of neural oscillations is known to shift during this period of development (impacting ERP amplitude differentially on the basis of age) I feel as though age needs to be included as a covariate in these correlations. I would recommend the authors run partial correlations with age as a covariate, using the raw behavioral scores and ERP data.

Can the authors explain why they elected not to include both RT and ERP in the same regression model?

Lines 343-344: The authors state that “children who showed high levels of learning, SES had a weaker effect 344 on grammar scores compared to children who showed low learning”. Can they provide the simple slopes (or similar) analysis and relevant statistics to support this claim?

Discussion:

The third paragraph of the discussion is very well-written, but I personally feel cuts short the impact of these findings! Can the authors please add a sentence or two that bolsters how stronger serial pattern learning skills may serve as a protective factor for grammar abilities in low SES children? I realize similar statements are included, but would love to see it more strongly stated as I feel this finding is not to be overlooked.

Thank you for your discussion of the null RT results in comparison to the ERP results.

In regards to grammar being significantly influenced by the interaction term, but not vocabulary, I appreciate the authors discussion and agree fully with the reasons they raise. Do they also believe the age of participants could be a factor? Did the groups significantly differ on vocabulary versus grammar? Could it be that ongoing development of complex grammar over this broad developmental time window introduces more variability, as compared to vocabulary (group means/SD would address this I believe)?

Thank you for your acknowledgement of the broad scope of ways SES may be measured.

**Minor:**

Introduction:

Lines 56-57: Can the authors expand on what they mean by “variations in language exposure” (i.e. conversational turns, quality/quantity of maternal input, etc.)?

Lines 92-96: I feel these lines are better suited for the methods section.

Methods:

Line 101 & 105: Please add the standard deviation of age

How many filler stimuli were present? How did this influence the embedded statistics of the task?

It is not entirely clear in the methods section that children are not tracking the colors, but that they are simply to press when food appears. The figure certainly helps with this, but could the authors explicitly state that the children were not pressing for a target hat, but rather when the food appeared, and that the hats were simply changing colors “in the background”?

Was there a training period?

Results:

Please italicize all *M/SD/p/N* etc. as per APA

Line 248: Why is RT mean, L-H, but ERP amplitude, H-L?

Line 250: What is the outlier labeling rule?

Line 289: Can you please include the two groups’ descriptive statistics created by the median split analysis? Also include group means/standard deviations on all behavioral measures.
