# Supplementary material for: How statistical learning interacts with the socioeconomic environment to shape children’s language development
Source: PLoS One. 2021 Jan 22;16(1):e0244954. doi: 10.1371/journal.pone.0244954 (PMC7822340; doi:10.1371/journal.pone.0244954)
Supplement: Attachment — Submitted filename: Manuscript reviewer comments-Final.docx [file pone.0244954.s002.docx]

We would like to thank the editor and reviewers for their thorough and helpful reviews. We would also like to thank the editor for giving us additional time to work on the revisions. We were happy to see that there was considerable enthusiasm for the first version of our manuscript and believe that we have addressed all of the comments raised in the reviews. The resulting manuscript is much improved and we thank you for considering it for publication. Below, our responses are in red font.

Reviewer #1. What a fantastic and timely paper! I am very excited about the findings presented in this manuscript. The authors make an excellent case for why this research is necessary, and the argument for investigating the interaction SES and serial pattern learning have upon language development is strong. I also greatly appreciate how straight forward and clear the writing is. That being said, I have some questions related to how the EEG data was analyzed that will potentially require major revision on the part of the authors.

**Major:**

General:

There are reports of a MMN difference for low versus high probability items in statistical learning paradigms/auditory oddball paradigms. Additionally, there are reports of N400/ERAN differences in similar paradigms. Given that other components could be present that could inform us of additional cognitive processes at play during learning, I am curious why the authors have focused their analysis only on the P300 component?

We acknowledge that different statistical learning tasks have been found to elicit a variety of ERP components. However, we have a priori focused on a late positive component in the time window and topography as outlined in the manuscript based on our previous work with this specific task and this general age range (e.g., Jost et al., 2015; Singh et al., 2018). We make this a priori prediction more explicit in the “Current Study” section of the Introduction. Note that based on the feedback from Reviewer 3, we realize that this late positivity does not match the typical characteristics of the P300 and therefore no longer refer to it as a P300 in the manuscript.

Introduction:

Lines 65-66: “Alternatively, it is possible that learning ability cannot compensate for such environmental limitations.” Do the authors feel it is possible that environmental factors could influence learning ability early on? For example, coming from a low SES home, where you receive poorer quality/quantity input, or poorer nutrition/higher stress/etc., could influence pattern learning?

We agree this is an important issue to consider. It is in fact one that we explicitly examined in our study. We found no effect of SES on statistical learning as measured with this particular task, as described in the results section.

Can the authors give a more detailed account of what serial pattern learning ability is, including background literature that links this specific type of learning to language development (with specific examples)?

We have opted to use the more established and commonly used term “statistical learning” throughout the manuscript. We have now added in additional review of findings from the literature that demonstrate a link between statistical learning ability and language development.

Additionally, can the authors include more concrete examples of how the P300 has been shown to reflect learning in similar paradigms? Specifically, discussing the previous studies (referenced in line 149) that lead the authors to predict faster reaction times and larger ERPs for HP than LP predictors.

We have added additional description in the “Current Study” section to illustrate how this late positive component has been shown to reflect learning using this paradigm.

Methods:

Measures:

Can the authors please include a breakdown of how many children were in each age group? Additionally, what was the mean age for each group in Table 1 (i.e. did age significantly differ across SES strata)?

We have included partial correlation analysis to control for age.

EEG:

In our prior draft, EEG method description had been streamlined but we realize we omitted important details. All method information has been added to or clarified in the “Electroencephalography (EEG) recording and processing” section on page **. All questions below about which acquisition and processing methods were used and the specific parameters of those methods are now described or clarified.

What was the sampling rate at collection? Was the data re-sampled?

Acquisition sampling rate was 250 Hz later resampled to 256 Hz.

Was the data low-pass/high-pass filtered?

The data were high-pass filtered at .1 Hz and low pass filtered at 100 Hz during acquisition. Data were later filtered offline during processing, high-pass at 1 Hz and low-pass at 30 Hz.

What was the reference electrode during collection? Was the data re-referenced offline?

The reference electrode was at the vertex during collection and re-referenced offline to the average reference.

How were segments of bad data removed? The authors state these were removed manually, but this occurs at the end of their EEG section, so did they remove them after already epoching the data and running ICA?

After filtering, resampling, and re-referencing, bad channels were removed and replaced with spherical interpolation of data. Then, ICA was performed and components with eyeblinks and eye movements were removed. After ICA, the continuous EEG was epoched and baseline corrected. At that point, further artifacts were removed manually.

The segment (epoch) length does not make sense to me. As I understand it, the stimuli was presented for 500 msec followed by a 500 msec black screen. This allows for a 1000 msec window per stimulus. Why would the authors epoch length then be 1500 msec with a 200 msec baseline? This includes the next predictor in the epoch—correct? Even if the authors are only going to investigate the first 500 msec of the epoch, additional noise is being introduced. If my interpretation is correct, I unfortunately would strongly recommend they epoch the data in a window that is more appropriate to their stimulus and time window of interest (-200 to 800 msec or something similar).

Thank you for this insightful suggestion. We took Reviewer 1’s advice and re-epoched the ERPs to be fully within the time of a single trial rather than overlapping with the following trial/predictor presentation. The epochs are now from 200 ms before the predictor is presented to 900 ms after it is presented on each trial (-200 ms – 900 ms). This is described in the “Electroencephalography (EEG) recording and processing” section on page **. All ERP processes that took place after epoching were redone and all analyses and plots involving ERPs were redone.

When the authors state that data were “replaced with data induced from surrounding sensors using the computational MATLAB software”, does this mean data was interpolated? Was this a spherical interpolation? Some other method?

A spherical interpolation was used.

How were ERPs created? Specifically, was the data low-pass filtered a second time? Was the data baseline corrected? Were single trials averaged together to obtain a stable waveform? Was this done for each condition and each electrode for every subject?

The data were low-pass filtered as second time at 30 Hz (and high-pass at 1 Hz as recommended for ICA by Winkler et al, 2015, [Winkler I, Debener S, Muller KR, Tangermann M. 2015. On the influence of high-pass filtering on ICA-based artifact reduction in EEG-ERP. Conf Proc IEEE Eng Med Biol Soc. 2015 Aug;2015:4101-5.](https://www.researchgate.net/publication/281290619_On_the_influence_of_high-pass_filtering_on_ICA-based_artifact_reduction_in_EEG-ERP)) The data were baseline corrected after epoching. Single trials were averaged together within condition within participant across the 6 electrodes in the region of interest. Each condition was then averaged over all participants.

Results:

I was surprised to see the authors analyzed the time window of 400-700 msec, as the P300 is traditionally 250-500 msec. Can this be considered the same effect?

As mentioned above, we have corrected our terminology and no longer refer to this as a P300 component as it does not fit the typical latency and topography characteristics of the P300. The 400-700 msec time window was selected a priori based on the previous research using this exact same paradigm with children of similar ages (Jost et al., 2015; Singh et al., 2018).

I greatly appreciate the authors inclusion of all data in Figure 4; however, this emphasizes to me that there are additional effects at play. A data driven approach, involving a cluster analysis (or some other variation where all time windows and electrodes are considered), would better allow for the investigation of all these effects. That being said, if the authors can make a stronger argument for a) only looking at the P300, b) only focusing on the 6 electrodes of interest and c) the time window of interest, I can also appreciate analyses that are rooted in solid a priori hypotheses. At this time though, those hypotheses (or why these choices were made) are not as clear as they need to be to not warrant a stronger, more data-driven approach.

As mentioned above, we have removed our discussion of the P300 and focused our discussion on the late positivity found with this statistical learning task in previous research (e.g., Jost et al., 2015; Singh et al., 2018). In addition, using criteria in articles suggested by Reviewer 3 (Keil et al., 2013; Luck and Gaspelin, 2017), we have clarified our argument for using the posterior region of interest. According to these guidelines, a priori selection of region of interest (as well as time window of interest) based on previously published research is the prefered method of selection. As reported in the original manuscript, we chose the posterior region based on findings from Jost et al. (2015) and Singh et al. (2018) who used an almost identical task and found differences between high and low predictor ERPs in a late positivity in the posterior region of interest. We have made this decision clearer in the current manuscript. After removing the perimeter (neck) electrodes (2 electrodes) from analysis due to EMG noise as well as sensors number 19 and 20 located directly behind the isolated common sensor (which often generates noise in the EGI nets), 6 electrodes remained in the posterior region. All were used for analysis.

Thank you for the Bonferroni corrections and for the inclusion of order (which turned out to be significant).

Correlation analyses: Given that the age range is very broad (7-12) and amplitude of neural oscillations is known to shift during this period of development (impacting ERP amplitude differentially on the basis of age) I feel as though age needs to be included as a covariate in these correlations. I would recommend the authors run partial correlations with age as a covariate, using the raw behavioral scores and ERP data.

Partial correlation analyses with raw behavioral scores and age as a covariate are now reported in Table 2

Can the authors explain why they elected not to include both RT and ERP in the same regression model?

We did not include both behavioral and ERP measures of statistical learning in the regression due to 2 reasons. First, we were interested in exploring distinct moderating effects of response time and ERPs on the association between parental education level and language measures. Second, we were concerned with an issue of power. We did not want to add an additional predictor variable to the same model. However, we are also aware of the risk of running multiple comparisons analyses on the same sample.

Lines 343-344: The authors state that “children who showed high levels of learning, SES had a weaker effect 344 on grammar scores compared to children who showed low learning”. Can they provide the simple slopes (or similar) analysis and relevant statistics to support this claim?

Thank you for bringing this up. We have added simple slope analyses to provide quantitative support for the reported claim on pages 16-17.

Discussion:

The third paragraph of the discussion is very well-written, but I personally feel cuts short the impact of these findings! Can the authors please add a sentence or two that bolsters how stronger serial pattern learning skills may serve as a protective factor for grammar abilities in low SES children? I realize similar statements are included, but would love to see it more strongly stated as I feel this finding is not to be overlooked.

We very much appreciate the reviewer’s opinion about the impact of these findings but want to be cautious and conservative in their reporting. We believe the current discussion does an adequate job of stating the impact of the findings without overstating them. If the editor feels differently about this, then we will be happy to add an additional sentence or two to the discussion as the reviewer suggests.

Thank you for your discussion of the null RT results in comparison to the ERP results.

In regards to grammar being significantly influenced by the interaction term, but not vocabulary, I appreciate the authors discussion and agree fully with the reasons they raise. Do they also believe the age of participants could be a factor? Did the groups significantly differ on vocabulary versus grammar? Could it be that ongoing development of complex grammar over this broad developmental time window introduces more variability, as compared to vocabulary (group means/SD would address this I believe)?

Thank you for bringing up this point. After refining the EEG processes (as recommended by the reviewers), we realized that 7-year-olds in our sample had the noisiest data and did not meet the minimum threshold for usable epochs in the analyses. Therefore, we removed them from further analyses. After doing so, we found vocabulary to be significantly influenced by the interaction term as well as grammar. This is explained in more detail under the “Moderation analyses” section. We have included group means and SDs in Tables 3 & 4. We also included age as a covariate in the regression models, and the interactions with grammar remained significant. However, after including age as a predictor in the model with vocabulary, the interaction was no longer significant. Interestingly, this may be related to the point you brought up regarding less variability in vocabulary with this age range.

Thank you for your acknowledgement of the broad scope of ways SES may be measured.

**Minor:**

Introduction:

Lines 56-57: Can the authors expand on what they mean by “variations in language exposure” (i.e. conversational turns, quality/quantity of maternal input, etc.)?

This is addressed in a clearer way.

Lines 92-96: I feel these lines are better suited for the methods section.

We moved this section to the Method section.

Methods:

Line 101 & 105: Please add the standard deviation of age

We added the standard deviation for age.

How many filler stimuli were present? How did this influence the embedded statistics of the task?

At the beginning of each trial, a random number between 1 and 7 filler stimuli were presented, then the predictor (or on occasion another filler), then either another filler (non-target) or the target was presented, and finally the trial ended with one more filler. From the participant's perspective, this was all continuous, so the filler at the end of one trial led directly to the first filler in the next trial. This created the impression of a continuous stream of fillers occasionally interrupted by another stimulus (predictor) that may or may not be followed by the target.

The number of fillers did not affect the relevant statistics for prediction as regardless of the number of fillers, the high predictor was followed by was followed by the target 90% of the time and the filler 10% of the time, while the low predictor was followed by the target 20% of the time and the filler 80% of the time. This has been made clearer in the “Statistical learning task” section on pages 11-12.

It is not entirely clear in the methods section that children are not tracking the colors, but that they are simply to press when food appears. The figure certainly helps with this, but could the authors explicitly state that the children were not pressing for a target hat, but rather when the food appeared, and that the hats were simply changing colors “in the background”?

Was there a training period?

This point has been clarified in the “Statistical learning task” section. High and low predictor and filler stimuli were indicated by color but target hats were the same color as filler. They were indicated by the presence of food.

There was no separate training period. However, the entire task could be thought of as “training” in the sense that it provided participants continual exposure to the statistical contingencies. There was no separate “test” phase. EEG was collected and measured continuously throughout as children were engaged in the target detection task and were (presumably) incidentally learning the relationships between the predictors and the target. Because of this, the continuous task was separated into 1st and 2nd half for analyses since our previous research has shown that children require time to learn the statistical contingencies (e.g., Jost et al., 2015). This has been clarified at the end of the “Statistical learning task” section.

Results:

Please italicize all *M/SD/p/N* etc. as per APA

We italicized these reported measures per APA.

Line 248: Why is RT mean, L-H, but ERP amplitude, H-L?

Participants took much longer to respond to condition H compared to condition L. We subtracted response times to H condition from response times to L condition to avoid reporting negative response times which would not be meaningful.

Line 250: What is the outlier labeling rule?

‘Outlier labeling rule’ is an approach for detecting outliers in normally distributed data by multiplying the first and third quartile of a distribution by a parameter (factor of 1.5 or 2.2). The resulting value will be added to the third quartile and subtracted from the first quartile values. This will define the boundaries of the "true" distribution and any values outside these boundaries are considered outliers. This was first introduced by Tukey in 1977 and revised by Hoaglin & Iglewicz,in 1987.

Line 289: Can you please include the two groups’ descriptive statistics created by the median split analysis? Also include group means/standard deviations on all behavioral measures.

We have included descriptive statistics created by the median split in Table 1. All group means and standard deviations for the behavioral measures are listed in Table 2

Reviewer #2: Thank you for giving me the opportunity to read this very interesting and well written paper on children’s processing of visual serial information and its relationship to SES. The paper raises two questions that are deeply related to questions around the impact of statistical learning (here referred to as visual serial processing) and language outcomes. The first asks whether statistical learning differences among children might relate to SES differences in language outcomes measured as vocabulary and grammatical learning. The second asks whether visual learning patterns might moderate the effects of SES. Using ERP and reaction time measures to a visual serial test. The authors find that that while there were no main effects of SES on these learning abilities, learning ability plus SES did provide some interesting relationship to language outcomes – here grammar. In particular, children with higher learning scores (measured through the reaction times for learning the probabilities in the statistical learning task) did better on grammar even if they were from low income homes.

The paper does an interesting job of relating aspects of language processing with aspects of language outcome in a statistical learning procedure. Yet, there were significant methodological problems with the manuscript to make me question the full force of the findings.

First, I did not understand why only 38 children were studied. Seems like a small N especially when broken down by SES and learning ability. Also about half of the children were black and I am hoping this is not a confound with SES though the paper does not allow me to tell this.

We have added this information in Table 1.

Second, the age band seems wide – 7 to 12 year olds? Why?

In looking at the data for re-epoching EEG data, as recommended by the reviewers, we noticed that the ERP waveforms for 7-year-olds were much noisier than those of the other participants (e.g.,in the previous draft, all participants who were excluded due to too few epochs remaining after removing artifacts were 7-year-olds). Thus, the revised age range reported in this study is 8-12. We have added justifications for using this age range in line 196-206.

We have added a statement about the possible confound of race or culture on p.29.

Third, there is other work on efficiency of processing which I would think is quite related to the work discussed here, but it is never cited. (see work by Fernald). There the authors do find the SES is directly related to the processing efficiency.

Thank you for bringing this to our attention. Now we have included the study by Fernald, Marchman, Weisleder (2013) on associations between processing efficiency and SES.

Fourth, I did not understand a fair number of the decisions on measures. For example, why would you measure learning ability on the same task that you measure visual serial outcome ability?

To clarify, there is one statistical learning task that includes both RTs and ERPs as dependent variables. These variables are used as our measure of “statistical learning” as reported in the manuscript.

And why do this when there were a host of other cognitive measures that would have allowed these to be treated independently? Unclear.

We apologize, but we are confused what is meant here. What other cognitive measures does the reviewer suggest to use instead of the statistical learning task we used?

And why only measure this in the second half of the task? To measure real abilities you would want to check out the slope of learning across both halves of the task rather than only when the students have learned the material.

We have clarified in the manuscript why we focus on the 2nd half of the task (because only with sufficient exposure to the statistical contingencies is learning observed, at least with the ERP measures, as described earlie). We report simple slope analyses results on pages 16-17.

Fifth, on line 85 I am told that past literature focuses on the P300 but the study then shifts by line 208 to 400 to 700. Why?

As mentioned previously, we have realized that we were in error in referring to the observed late positive component as a P300. Therefore, we do not discuss the P300 literature and instead focus on previous research that has also observed this component using the same paradigm.

Sixth, I worried that the statistical treatment was all over the place and largely done to reveal findings rather than to do a comprehensive look at the data. Sometimes ANOVAs are used, sometimes continuous variable assessments like multiple regression, sometimes independent t-tests (line 287) and sometimes visual inspection (line 207). I should think you would want a comprehensive way to explore the data with very clear rationale and precedence for the decisions made.
Thank you for bringing up this concern regarding the use of various statistical analyses in our study. We have included a detailed rationale for using each specific analysis.

In short, this is a potentially interesting question that leaves the reader wondering why certain choices were made in sample, methods and analyses and without a better understanding of these variables, it is hard to know how to interpret the findings in a coherent way. As of now, even what is reported seems a tad stretched –statistical learning patterns are related to language outcomes for children who learning them better, but only for language outcomes in vocabulary and only when measured after they have some experience with a task.

Reviewer #3: 1. The introduction is very short and lacks clear hypotheses. Requires a more detailed literature review of the P3, and why it is the component of interest here. Previous work has identified other components in serial tasks such as this, so it’s not clear why the P3 is the focus (especially since the results do not look anything like a P3).

As mentioned previously, we have expanded the introduction by providing more description of previous research linking statistical learning to language development, as well as providing more details about the learning paradigm we are using and some of the research findings derived using this paradigm. Also as mentioned, we no longer discuss the P300 as the late positivity of interest does not appear to be one. We also have added hypotheses for the expected ERP effects as well as hypotheses for the two primary research questions of the study.

2. Further to that point, and perhaps my biggest concern with this paper: the ERP effect you report looks nothing like a P3 in either the waveform plots or scalp maps. A P3 typically peaks around 300 ms (although there is variance), looks like a single waveform peak, and is maximal over Cz/Pz. Your effect is significantly later in time, looks more like a plateau than a peak, and has a scalp distribution in which the positivity is largest over lateral electrodes. As noted below, it appears you used CZ as a reference so it’s possible that this will look more like a P3 with appropriate re-referencing. But regardless, you should (a) in the Introduction clearly describe the predicted characteristics of the P3, supported by citations (ideally in studies as similar to yours as possible, since task and stimulus parameters will influence how it looks), and (b) in interpretation, provide a rationale for interpreting your effect as a P3, or consider that this may not actually be a P3 and if so, what it might be instead.

We thank you for pointing out our error in interpretation in the previous draft. We no longer characterize this component as a P300 for the reasons you give. Instead, we focus our review and predictions based on this same late positive component that was elicited using the identical learning paradigm with children of similar ages. As discussed in the “Current Study” section, this component appears to reflect learning of the predictor-target contingencies and may index an awareness that the predictor signals the upcoming occurrence of the target stimulus (i.e., a form of predictive processing). (e.g., Jost et al., 2015; Singh et al., 2018).

3. The hypothesis that “we expected to observe a P300 effect reflecting learning,” (line 85) is insufficient in detail. What is the nature of the predicted P300 effect? Will it be larger or smaller after learning, and how in relation to your experimental conditions? Obviously, this also necessitates describing your experiment in sufficient detail in the introduction that the reader understands what the experimental conditions are.

We now describe the paradigm in more detail in the “Current Study” section and are more explicit about the nature of the ERP effects and how they relate to learning performance.

4. As well, it’s insufficient in the introduction to simply list the behavioural methods that you included. Please be clear on how you expected these to relate to the ERP effects and behavioral learning effects, again in terms of things like larger/smaller P3/RTs to condition X relative to condition Y.

This has been clarified/added.

5. The rationale that is provided for the P3 is weak. A “shift in attention to stimuli…” (line 90) doesn’t make sense because in your study, there is no attention shifting – there is a single stimulus on the screen at a time and subjects are expected to be attending to all.

6. The manuscript contains no indication that the study complied with relevant ethical guidelines. In the Participants section please clarify whether participants provided informed consent, whether an ethics board reviewed the study, and whether the practices were in compliance with the Declaration of Helsinki.

We had included in an earlier version of the manuscript that we collected written informed consents and assents from the caregivers and participants, respectively. We apologize that this information was omitted in the version submitted. We have revised the section to include IRB approval and compliance with ethical guidelines in the Declaration of Helsinki.

7. In Methods, please clarify what was used as your SES measure. This becomes clear only later. You do state “We used the average of both caregivers’ education level as a measure of SES.” (line 115) but in saying “a” measure and not “the” measure, it leaves ambiguity.

Thank you for bringing this up. We have fixed this error.

8. Further to the above point, in the Results section it is misleading to refer to your measure of average parental educational level as SES. Please refer to it as “parental education level”. In the Discussion you can generalize as SES – but please again include the caveat that in this case you used education as a proxy for what is typically described as SES, which includes income and types of employment held by the parents.

We agree that we should not have generalized our results with parental education level to the overarching construct of SES. We have replaced “SES” with “parental education level” in the Method and Results sections to address this issue.

9. Learning Task (Methods): please clarify the number of filler trials included

There were 60 filler trials, half with a target and half without. This information has been added to the “Statistical learning task” section on pages 10-12.

10. A hypothesis appears in the methods on line 150. Hypotheses should be in the Introduction, not buried in the Methods

The hypotheses are now clearly stated in the introductory “Current study” section.

11. EEG analyses are a problematic area. First off, as noted by another reviewer, you provide insufficient detail on the EEG methods and analysis. You should read and follow published guidelines for ERP studies:
Keil, A., Debener, S., Gratton, G., Junghöfer, M., Kappenman, E., Luck, S., Luu, P., Miller, G., Yee, C. (2013). Committee report: Publication guidelines and recommendations for studies using electroencephalography and magnetoencephalography Psychophysiology 51(1), 1 21. <https://dx.doi.org/10.1111/psyp.12147>

Thank you for pointing us toward that article. We did follow the recommendations of that article, but failed to describe how we followed them. We read the article and incorporated discussion of how we followed the guidelines and referenced the article in appropriate places in the manuscript. All details requested by Reviewer 1 were included in the “Electroencephalography (EEG) recording and processing” section on page 14.

12. In particular, please (at least) provide the sampling rate used during data collection, ALL preprocessing steps performed, the name and version of software used (appears to be EEGLAB, but “Matlab” is insufficient). Also clarify the method used for electrode interpolation.

All of this has been included in the “Electroencephalography recording and processing” section.

13. What was the reference electrode used during recording? Was the data re-referenced during preprocessing? If not, this is a significant problem that likely influenced your results. The EGI system by default uses Cz as the reference during recording. Cz is around where the maximal P3 effect would be observed, so in using this as reference you would effectively subtract the P3 from your data. This indeed is what is suggested by inspection of your scalp topography maps. If you are unaware of issues around re-referencing, you should consult Luck’s book or (shameless self-referencing) my recent book “Research Methods in Cognitive Neuroscience” which addresses this issue in detail, with examples.

We did acquire the EEG with a vertex/Cz reference electrode; however, we did re-reference to the average reference. This information has been added to the “Electroencephalography recording and processing” section.

14. Please also read and consider Luck, S., Gaspelin, N. (2017). How to get statistically significant effects in any ERP experiment (and why you shouldn't) Psychophysiology 54(1), 146-157. <https://dx.doi.org/10.1111/psyp.12639> - your analysis appears to follow many of the “worst practices” highlighted there, notably post hoc selection of electrodes and time windows based on visual inspection. This is particular true of the time window – ayone predicting a P3 on the basis of the literature would focus on an earlier time window than 400-700 ms, whereas this is very obviously the time window one would pick based on post hoc visual inspection of the data.

Thank you for this reference. In our previous draft, we apparently were not clear that our selection of electrodes and time window was not in fact post-hoc but was an *a priori* decision based on the literature using this specific paradigm (e.g., Jost et al., 2015). We believe the current manuscript makes this point clearer. A priori is the preferred method of choosing regions of interest and time windows. We have added the Luck & Gaspelin reference to the manuscript in the “Electroencephalography recording and processing” section.

15. There are some systematic problems with how statistics are reported and interpreted. For one, the treatment of interactions is incorrect. For example, in the behavioral results section you report an interaction of predictor condition and block. However, the follow-up statistics you provide do not fully test the interaction; you report comparisons of condition separately in the first and second blocks, but then only qualitatively compare the magnitude and direction of these differences between blocks. Properly explaining the interaction requires that you statistically compare the condition difference, between blocks. The same is true for your ERP analysis. As it turns out, this was done, but it is reported later in section 3.3. This section should be removed because what is contained there are really the analyses you should be presenting to interpret your interactions.

Thank you for this feedback. We have clarified our interpretation of the interaction between block and condition on lines 356-372 for RT and lines 408-424 for ERP data.

16. Incidentally, section 3.3 (line 247) has inconsistent heading formatting -it is the only section with a numbered heading and is nested under “neurophysiological evidence of learning” even though it does not exclusively relate to ERP evidence).

We have fixed this error in formatting.

17. Lines 223-235 present a qualitative description of scalp distributions of the effects. This includes evaluative language like “the voltage response in posterior regions is stronger”. This is not appropriate. If you wish to make such claims you need to support them quantitatively with statistical analyses.

This language has been removed.

18. Please indicate in the legends for Figs 5-6 what the error bars represent (95% CIs? SE?)

We clarified this for figures 5 and 6.

19. Figure legends are poorly formatted. They are inline with the body text of the manuscript which makes reading the text hard. As well, since the figures appear at the end, it is very hard to interpret the figure due to the need to scroll back and forth between legends and figures. Please provide legends immediately before figures as is standard practice.

We had placed figure legends inside of text and all figures at the end of the manuscript as required by PlosOne submission guidelines. We agree on the importance and convenience of placing legends and figures together in any type of writing. We will work on formatting tables and figures in accordance with standard practice and submission guidelines from PlosOne.

20. Please provide a rationale for describing your RT and ERP results as reflecting learning “ability”. It seems that you have a single measure of learning and are generalizing from this to a more generalized “ability”.

We have changed the wording throughout to be more precise about what the RT and ERP variables are measuring. In most cases we now refer to this as statistical learning “performance” or “scores”. However, in the discussion and abstract, we occasionally retain the term “ability” because we believe it is not improper to use the particular measures and results from this task to generalize to the larger construct of statistical learning.

21. Correlation analyses: You treat SES as a continuous variable in the correlation analyses. I’m not convinced this is appropriate. The measure is not continuous, it’s ordinal. Furthermore, contra the assumption of a linear measure, the conceptual “distance” between the levels of the SES variable are not necessarily even steps. For instance, is the difference in SES for parents with a PhD relative to those with a professional degree really the same as between high school and some college? See, for example, <https://www.frontiersin.org/articles/10.3389/fams.2017.00015/full> for a description of the issue. This is an area in which there is a lack of consensus, and in many cases the linearity assumption is approximately correct. However, you need to explicitly recognize and address this issue in your manuscript, and justify treating SES as continuous. See, e.g., <https://www3.nd.edu/~rwilliam/stats3/OrdinalIndependent.pdf>.

Thank you for these helpful links. This is a very important point that we need to include in reporting these results. Each caregiver’s reported education level was ordinal. Then we took the average of the 2 education levels (1 from each parent/caregiver) which created a continuous variable. We have added a statement addressing this issue in our Discussion section. Furthermore, we combined the data across “PhD” and “Professional degree” categories and created the “PhD/Professional degree” category in an attempt to assimilate variations between categories in this variable.

22. Please clarify whether multiple comparison correction was used in computing the significance of the 45 correlation values reported in Table 2

We added correlation results that were corrected for multiple comparisons in Table 2.

23. The “influence of SES on learning” section applied a median split to the SES data. First off, please clarify what cutoff this resulted in – from Table 1 my guess is between 3 and 4 on your scale, but this should be made explicit. Assuming this is so, an important thing to address in your Discussion is that this analysis differentiates between people with college degrees (bachelor’s or higher) and those without. As noted above, this is rather different from a more general construct of “SES”

We addressed both of these points in our Discussion section

24. Discussion: Around line 404, consider the alternative explanation that in addition to “making a prediction about the upcoming stimulus”, your ERP effects reflect response preparation.

This point was added.

25. Line 448: You state, “…the lack of a significant correlation between SES and learning ability and the non-significant effects of SES on learning in the present study suggests that what drives variation in learning is not due to social environmental factors such as SES”. You are overstepping your data here, in the last part of the sentence. Please remove “social environmental factors such as” and just say SES, as you have no evidence on other social-environmental factors to justify this claim.

We fixed that error in our discussion section.

26. Lines 464-465: please quantify the proportion of subjects in each SES group by race (either here or earlier when the SES distribution is reported)

This information is added. We reported this in Table 1.

27. Lines 473-476: You suggest that serial pattern learning abilities might help “ameliorate language disadvantages”, but elsewhere my interpretation of your writing was that you thought that relationships between grammatical and serial learning abilities are because they rely, at least in part, on the same neurocognitive machinery. If that is so, then why do you imply a unidirectional influence of serial learning abilities on grammar? Would grammar training not be equally predicted to boost serial learning abilities?

Thank you for this insight. We agree that it is theoretically possible that training of grammar could improve statistical learning. On the other hand, while there exists longitudinal data showing that statistical learning predicts language scores later in life, no longitudinal data, that we know of, suggests that grammar scores predict statistical learning. In addition, the statistical learning literature generally treats this ability as the more basic mechanism that allows for language to develop, rather than the other way around. Thus, although there may be a bidirectional effect between statistical learning ability and grammar, we are not aware of any studies reporting such findings, and believe that the extant literature is more supportive in treating statistical learning as the causal factor affecting language development.
