# Supplementary material for: How statistical learning interacts with the socioeconomic environment to shape children’s language development
Source: PLoS One. 2021 Jan 22;16(1):e0244954. doi: 10.1371/journal.pone.0244954 (PMC7822340; doi:10.1371/journal.pone.0244954)
Supplement: Attachment — Submitted filename: Manuscript reviewer comments-V2_Final.docx [file pone.0244954.s003.docx]

We would like to thank the editor and all reviewers for their helpful feedback which has greatly improved this manuscript. We believe we have addressed all of the reviewers’ comments. Please find our responses in red.

Reviewer #3: # Conway PONE

1) P. 7 Please change the word “impairment in the sentence, “low SES may be associated with impairments to a variety of cognitive abilities including working memory, cognitive control, and language”. The word “impairment” is both loaded, and inaccurate. The citation used to support this statement does not contain the word stem “impair” anywhere in it, and indeed talks of the effects of SES in terms of correlations and gradients, and notes that “…the statistical effects of SES operate across the whole SES continuum…” with little evidence of “threshold” effects. Please use more graded language, such as “low SES may be associated with lower scores on a range of measures of cognitive abilities…”
Thank you for bringing this up. We have incorporated this change.

\## Hypotheses:
2) - Hypotheses are improved but still need work. There is no mention of ERPs with respect to the two research questions of the study. That is, there is no prediction as to how SES will relate to ERPs. If SES-related questions are the primary research questions of the study, and no predictions about EPRs were made, this begs the question of why ERP data was collected or presented. Please state predictions regarding ERP data with respect to SES.

The previous several paragraphs described in detail how the ERPs (and RTs) are being used to operationalize learning in the task. Therefore, we thought it was clear that the predictions/hypotheses relate to these measures of learning. However, we have added clarification to the hypotheses.

\## Methods
3) Data from 7 year olds was collected as part of this study, and was included in the first draft. For this second draft, the authors have chosen to remove these data due to low trials/noisiness. While this rationale is acceptable, removing all mention of these participants, and why their data was removed from the analysis, is not. This is not open and transparent scientific practice. Indeed, the statement on p. 7 “We recruited 27 typically developing, monolingual English-speaking children aged 8-12 “ is entirely false, given that the previous version of the manuscript stated, “We recruited 42 typically developing, monolingual English-speaking children aged 7-12…” Moreover, the following sentences in this section provide a rationale that misleadingly implies that the 8-12 year old range was planned a priori (“We chose the age range 8-12 years for four reasons…”). Please include mention of the 7 year olds in the methods and provide your rationale there for removing them from analysis.
Thank you for raising this valid point regarding reporting of the participants. We revised this section as you suggested by reporting the age of the original sample and explained why the 7-year-olds were excluded from the analyses.

\### SL Task
4) I was confused by the statement, “Each experimental condition (HP and LP) contained 60 trials.”. It would be helpful to the reader if you moved the paragraph starting with the words, “Each stimulus was presented for 500 milliseconds…” on p. 11 up to appear after the sentence, “The participants viewed a stream of flashing stimuli consisting of hats of different colors presented one at a time with a black background.” On p. 10, or somewhere around there.
We have restructured this section based in part on your suggestion. Hopefully the changes improve the readability and clarity of the task description.

\### EEG preprocessing
5) The authors report 1 Hz high-pass filtering of there EEG data. This seems excessive for EEG data, especially in light of evidence that even 0.3 Hz filtering can attenuate real ERP components and induce artifactual ones (Tanner, D., Morgan‐Short, K., & Luck, S. J. (2015). How inappropriate high‐pass filters can produce artifactual effects and incorrect conclusions in ERP studies of language and cognition. Psychophysiology, 52(8), 997-1009.). Is this a typo, or can a rationale for this extreme filtering be provided?
This was a typo! The correct high-pass filter is .1 Hz. Thank you for catching this error.

6) p. 13, line 7: don’t capitalize “all” after the semicolon

We corrected this error.

7) p. 13, para 2, line 2 - require period after Jost citation.
We corrected this error.

8) p. 13, again after a Jost citation a period (and space) are missing, in the sentence ending “…also an a priori decision based on the findings from Jost et al. [56]”

We corrected this error.

\## Results
9) The reporting of behavioural (RT) results is confusing and at times inaccurate.
(a) The first paragraph of the results section mentioned a condition x block interaction, but then provides follow-up t-tests that don’t really support or explain the interaction: the fact that RTs were significantly faster for HP and LP in both blocks is not evidence for an interaction, it’s evidence for a main effect of condition.
(b) The next paragraph refers to “the” main effect of block, as if this had been previously reported (and providing no statistical information to support the claim of significance).
(c) Further, the t-tests used to address whether there was an ME of block aren’t appropriate for that question: they report the differences between blocks separately for HP and LP conditions. By definition, a main effect of block is _collapsed_ across levels of any other condition. The appropriate way to report this is to report the F and p values from the ANOVA for the ME of block. The authors state that this was not significant, so upon providing the F and p values to back up that claim, no subsequent t-tests are necessary.
(d) Further, the main effects of the ANOVA (for both block, and condition; the latter is currently missing) should be reported first, and the interaction second. Note that t-tests appropriate to explaining the interaction are provided, in the latter part of paragraph 2. These need to follow after the reporting of the interaction in the ANOVA
Thank you for your feedback and suggestions. These issues have been fixed.

10) Figure 3’s caption needs further detail. The bars are surely indexing the _difference_ in RT between HP and LP conditions, in each half of the experiment, but this is not stated in the caption. Please clarify this fact in the caption.

We clarified this point in the caption for figure 3.

11) Similar errors in ANOVA reporting are present in the ERP analysis, as in the RT analysis. Specifically, the third line from the bottom of p. 17 starts, “Additional paired sample t-tests exploring the main effect of block…” and then reports t-tests computed separately for the HP and LP conditions. Again, an analysis of block that is broken down by condition is not a test of the main effect of block, it is another way of examining the block X condition interaction. This section goes on to conclude, on the basis of these t-tests, “These results suggest that there is no main effect of predictor condition and block…” - which is again not the way to assess the ME of either condition or block. Please simply report the statistical values (F and p) for the main effects of each factor, that were provided by your ANOVA results.

These issues have been fixed.

12) the motivation for the median-split comparisons of high vs. Low SES groups on ERPs and RTs is not clear. The lack of a relationship between these variables was already established by the correlation results in the previous section. Please provide a rational or remove these effectively redundant analyses.

We removed the redundant analyses from this section and made a reference to the correlation table.

13) The statement in the first line of the last paragraph of p. 21 is conducing and misleading. It states, “…results of the regression analysis using the neurophysiological measure showed that statistical learning ability was a significant moderator of the relationship between parental education and both language measures”. My issue is that the authors are defining a difference in ERP amplitude between the HP and LP conditions as “statistical learning ability”. I’m not convinced that an ERP difference reflects an “ability”, especially in the absence of a parallel significant moderating effect of the behavioral measure of statistical learning. I suggest that the authors use a more neutral and empirically-based term here (e.g., “difference in ERP amplitude”) and provide a interpretation/rationalization in the discussion as to why they equate differences in the ERP amplitude with differences in learning ability.
We have removed the term “statistical learning ability” and instead use the term “statistical learning performance”. However, we feel it is appropriate to use the term “ability” in the Discussion section as we are assuming that performance on this task does tap into an underlying learning ability. We clarify upon first use of the term “ability” in the Discussion section that it is based on the ERP and RT performance data.

14) p. 24 line 1, please remove the space after “however” before the comma

We corrected this error.

15) p. 24 line 3, “As an additional control…” should be the start of a new paragraph

We corrected this error.
